# Economic costs of fever to households in the middle belt of Ghana

**DOI:** 10.1186/s12936-016-1116-x

**Published:** 2016-02-06

**Authors:** Theresa Tawiah, Kwaku Poku Asante, Rebecca Akua Dwommoh, Anthony Kwarteng, Stephaney Gyaase, Emmanuel Mahama, Livesy Abokyi, Seeba Amenga-Etego, Kristian Hansen, Patricia Akweongo, Seth Owusu-Agyei

**Affiliations:** Kintampo Health Research Centre, P. O. Box 200, Kintampo, Ghana; London School of Hygiene and Tropical Medicine, London, WC1E7HT UK; School of Public Health, University of Ghana, Accra, Ghana

**Keywords:** Malaria, Treatment-seeking behaviour, Direct cost, Indirect cost, Kintampo, Ghana

## Abstract

**Background:**

Malaria is one of the main health problems in the sub-Saharan Africa accounting for approximately 198 million morbidity and close to 600,000 mortality cases. Households incur out-of-pocket expenditure for treatment and lose income as a result of not being able to work or care for family members. The main objective of this survey was to assess the economic cost of treating malaria and/or fever with the new ACT to households in the Kintampo districts of Ghana where a health and demographic surveillance systems (KHDSS) are set up to document population dynamics.

**Methods:**

The study was a cross-sectional survey conducted from October 2009 to July 2011 using community members’ accessed using KHDSS population in the Kintampo area. An estimated sample size of 4226 was randomly selected from the active members of the KHDSS. A structured questionnaire was administered to the selected populates who reported of fever within the last 2 weeks prior to the visit. Data was collected on treatment-seeking behaviour, direct and indirect costs of malaria from the patient perspective.

**Results:**

Of the 4226 households selected, 947 households with 1222 household members had fever out of which 92 % sought treatment outside home; 55 % of these were females. 31.6 % of these patients sought care from chemical shops. A mean amount of GHS 4.2 (US$2.76) and GHS 18.0 (US$11.84) were incurred by households as direct and indirect cost respectively. On average a household incurred a total cost of GHS 22.2 (US$14.61) per patient per episode. Total economic cost was lowest for those in the highest quintile and highest for those in the middle quintile.

**Conclusion:**

The total cost of treating fever/malaria episode is relatively high in the study area considering the poverty levels in Ghana. The NHIS has positively influenced health-seeking behaviours and reduced the financial burden of seeking care for those that are insured.

## Background

Malaria is one of the main health problems in sub-Saharan Africa [1]. In 2013, there was an estimated 198 million cases of malaria with estimated deaths of 584,000 in Africa [2]. The annual reported confirmed malaria cases in Ghana in 2013 were about 1.6 million out of which 2506 resulted in deaths. In addition to being responsible for a high rate of morbidity and mortality, malaria can lead to impaired cognitive development and learning abilities [3], school absenteeism [4] and low levels of attention at school [5].

Malaria and its associated costs present a major socio-economic challenge to households in Africa. It leads to considerable economic burden at the household level and the poorest households in the population are mostly affected [6, 7]. Households are affected through out-of-pocket expenditure for treatment and by the loss of income as a result of not being able to work or care for family members. Cost of malaria constitutes significant shares of annual household incomes ranging from 6 to 13 % depending on the burden of malaria [8, 9]. Cost of time lost to patients and caretakers as a result of the disease constitute more than fifty percent of total cost of treating malaria [6, 7]. Loss of working days due to malaria by a sick adult in some malaria endemic areas of Africa has reported a range between 6–9 days. This leads to substantial loss of productive man-hours to households, resulting in decrease in household income [6, 10–12]. Malaria in Africa has been reported to cause about 1.3 % reduction in annual economic growth rate [10, 13, 14].

WHO recommended treatment of malaria with combination therapy preferably those which contain artemisinin [15] for countries suffering from malaria. This is because artemisinin-based combination therapy (ACT) has been known to be safe and effective with known slow emergent resistance by malaria parasites. Ghana introduced ACT as the first-line drug for the treatment of uncomplicated malaria in 2004 [16]. Though ACT is safe and effective compared to monotherapies, it is however much more expensive [17]. This survey sought to assess the economic cost of treatment-seeking for malaria and/or fever with the new ACT to households in the Kintampo municipality of Ghana. This study is part of INDEPTH Phase IV Safety and Effectiveness Studies (INESS) carried out between 2009 and 2011 as part of assessing the real life determinants of effectiveness of new anti-malarials that are introduced after licensure [18].

## Methods

### Study area

Kintampo districts cover an area of approximately bout 7162 km^2^ a resident population size of about 150,000 inhabitants in 29,438 households [19]. Kintampo Health Research Center (KHRC) located in the Kintampo districts maintains the Kintampo Health and Demographic Surveillance System (KHDSS), which was used for sampling households for this study. The KHDSS routinely collects demographic information on age, sex, pregnancies, births, deaths, and migrations among others and does verbal autopsies to determine the cause of deaths [20]. The site also collects data on household assets and possessions that enable the assessment of socio-economic status of the population. There are two hospitals, twenty health centres, three private clinics and five public Community Health Planning Services (CHPS) compound in the study area. At the time of this study about 50 % [21] of inhabitants in the study area had valid National Health Insurance cards to access health care in public and private accredited by the National Health Insurance Authority (NHIA). Currently, health insurance coverage is about 66 % [22]. The study area is mainly rural with few urban settlements. Farming is the main economic activity in the area. Malaria accounts for more than half of all out-patient visits in the study area [23].

### Study design

This was a cost analysis study based on patient’s perspective. A cross-sectional household survey was conducted between October 2009 and September 2011 among community members in the Kintampo area located in the middle part of Ghana.

### Participant selection and data collection

Assuming 6 % of the entire inhabitants will have had fever in the 2 weeks prior to the day of interview and that 50 % of them will seek care and have physical access to an authorized point of provision of ACT: then with 95 % confidence and allowing for 10 % drop-out, the estimated sample size of 4226 households will be achieved. Households were randomly selected from the 4226 households using the KHDSS household database and interviewed with a structured questionnaire. Household members who reported of fever within the last 2 weeks prior to their interview were included in the survey. However, only those who sought treatment outside their homes and incurred costs in seeking treatment were included in the analysis. Data was obtained from household members on socio-demographic characteristics, treatment-seeking patterns as well as direct and indirect costs of seeking treatment outside home.

### Data management and analysis

Data was double-entered into Epidata 3.1 (THE EPIDATA ASSOCIATION, ODENSE M, DENMARK, EUROPE) and transferred to Stata 11.0© (STATA CORP. TX) for analysis. The total economic cost of fever per household was estimated by summing the direct and indirect costs incurred per household. The estimated mean and median costs with their respective 25th and 75th percentiles (iqr) and standard deviations (SD) are presented. Direct costs were defined as out-of-pocket expenditure incurred by the households as a result of seeking treatment for their fever outside their homes. It included cost of self-treatment, medical costs and non-medical costs. Cost of self-treatment comprised the cost of medicines purchased from pharmacies, chemical and other drug sellers without prescription from a doctor or medical staff at a health facility. Medical costs included consultation, laboratory and prescription fees incurred at a health facility. Non-medical cost included cost of transportation to and from the facility where care was sought and other non-medical costs incurred as a result of seeking health care for the treatment of fever.

Indirect cost was defined as the cost of productivity losses to households as a result of their fever and inability to work. It was calculated as a product of the number of days respondents were unable to work and their daily wage. Daily wage was estimated in several ways depending on the type of economic activity the respondent was involved. The daily wage for economically active adults (defined as persons aged 18 years and above; and engaged in informal income generating activities, such as farming) who hired others to do their work during the course of the fever was calculated as the cost paid out to others to get the work done. Among patients who earned monthly income, daily wage was estimated as their total monthly income divided by 22 days with the assumption that they worked 5 days per week for a month of 4 weeks. Students were asked to report on the number of school days missed due to fever. When adult patients were accompanied by caretakers, the daily wage of their caretaker was estimated in a similar manner for the patients. Seeking care outside home was defined as seeking care from the public or private health facilities, chemist or pharmacist and herbalist or drug peddler.

Wealth quintiles for each household were calculated using Principal Component Analysis (PCA) and included durable households’ possessions such as motorbike, car, bed, radio and sewing machine. Information on household architecture of material for walls, roofing material, source of drinking water, cooking utensils, toilet facility and cooking fuel [24–26] were also obtained. Exchange rate (as at 30th September 2011): GHS 1.52 is equivalent to $1 [27].

### Ethical issues

Written informed consent was obtained from all adult participants and from care-takers of children. Additionally, assent was sought from participants who were between 12–18 years. The Kintampo Institutional Ethics Committee and the Ghana Health Service Ethical Review Committee granted ethical approval for the study.

## Results

### Socio-demographic characteristics

Of the 4226 households selected, 947 (22.4 %) households with 1222 household members reported of having fever within the 2 weeks prior to their interview. Ninety-two percent (1127/1222) sought treatment outside their homes and, therefore, met the criteria for inclusion in the analysis. More females reported of fever than males (Table 1). Fifty-eight percent of the patients were below 18 years of age. Fifty-four percent and 15.1 % of those who reported of fever were farmers and unemployed, respectively. About 17.4 % of the respondents were in the highest wealth quintile whilst 21.2 % were in the lowest wealth quintile (Table 1).

**Table 1:** Socio-demographic characteristics

Variables	Total number	Percentage (%)
Had fever and sought care outside home	1127	92.2
Response rate		
Consent given	1127	100
Age groups (years)		
<18	658	58.4
≥18	469	41.6
Sex		
Male	503	44.6
Female	624	55.4
Main occupation		
Farmers	255	62.7
Formal sector	33	8.1
Traders	84	20.6
Artisans	25	6.1
Unemployed	9	2.2
Other	1	0.3
Wealth quintiles		
Highest	194	17.2
Fourth	223	19.8
Middle	278	24.7
Second	193	17.1
Lowest	239	21.2

### Households’ treatment-seeking behaviour

Close to 32 % (356/1127) of patients who sought care outside their homes did so from chemical shops, 22.7 % (256/1127) from hospital, 17.9 % (202/1127) from private clinics and less than 5 % from Community based Health Planning and Services (CHPS). Majority of respondents in the different wealth quintiles sought care from the chemical seller shops. Most of the respondents in the rich group sought care from the private clinics, as 39 % of patients in the highest wealth quintile sought care from private clinics compared with only eight percent in the middle class who sought care from private clinics. Majority of respondents, who sought care from the CHPs compounds were from the poorest of the population. Respondents who sought care from drug peddlers were mostly from the lowest socioeconomic groups (Table 2).

**Table 2:** Treatment-seeking behaviour

Name of provider	Number (%)	Wealth quintile groupings				
		Highest (%)	Fourth (%)	Middle (%)	Second (%)	Lowest (%)
Chemical seller Shop	356 (31.6)	53 (27.3)	64 (28.7)	109 (39.2)	51 (30.8)	69 (29.7)
CHPs	45 (4.0)	1 (0.5)	9 (4.0)	8 (2.9)	14 (7.3)	13 (5.4)
Private Clinic	202 (17.9)	76 (39.2)	52 (23.3)	23 (8.3)	21 (10.9)	30 (12.6)
Drug peddler	90 (8.0)	0 (0.0)	5 (2.2)	30 (10.8)	40 (20.7)	15 (6.3)
Herbalist	10 (0.9)	2 (1.03)	1 (0.5)	2 (0.7)	4 (2.1)	1 (0.4)
Health Centre	144 (12.8)	5 (2.6)	20 (9.0)	48 (17.3)	25 (13.0)	46 (19.3)
Hospital	256 (22.7)	49 (25.3)	64 (28.7)	56 (20.1)	28 (14.5)	59 (24.7)
Pharmacy	24 (2.1)	8 (4.1)	8 (3.6)	2 (0.7)	2 (1.0)	4 (1.7)
Total	1127 (100)	194 (100)	223 (100)	278 (100)	193 (100)	239 (100)

### Households mode of payment for cost of fever

**Fig. 1:**
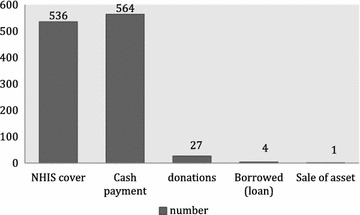
Mode of payment of cost of fever

Households used different means to pay for the cost of fever (Fig. 1). About 48 % of respondents (536/1132) had their cost of fever covered by the National Health Insurance Scheme (NHIS); 564 (49.8 %) respondents paid cash for the care of fever; a minority of respondents paid for their fever through donations, borrowing and sale of their assets.

### Days lost due to fever and to productivity

A total of 2020 days with an average of 4.2 days per patient was lost due to the reported fever cases (Table 3). Sixty-five percent (252/386) of students were not able to attend school due to fever leading to a total of 904.4 school days lost and an average of 2.4 days lost per pupil/student. For adults, the average number of days lost due to fever was highest among farmers (6.4 days) and lowest among formal sector workers (5.3 days) (Table 3). The average number of days lost per patient was highest among those who sought care from herbalist (6.9 days) and lowest among those who sought care from private clinics (3.4 days) (Table 4).

**Table 3:** Days lost to productivity by socio-demographic groups

Variables	N	Total days lost	Mean days lost	SD	Median	iqr (25th–75th percentile)
Age groups (years)						
<18	40	36	0.9	2.9	0	0
≥18	441	1984.5	4.5	4.3	4	0–7.0
Total	481	2020.5	4.2	4.3	3.0	0–7.0
Sex						
Male	167	768.2	4.6	4.4	4.0	0–6.0
Female	314	1256	4.0	4.2	3.0	0–7.0
Total	481	2024.2	4.2	4.3	3.0	0–7.0
Students	386	937	2.4	2.6	2.0	0–4.0
Main occupation						
Farmer	253	1442.1	5.7	4.2	5.0	3.0–7.0
Formal sector	27	94.5	3.5	4.1	3.0	0–6.0
Trader	86	361.2	4.2	4.2	3.0	0–6.0
Artisans	25	92.5	3.7	4.2	4.0	0–5.0
Unemployed	9	14.4	1.6	2.5	0	0–2.0
Other	1	6	6.0	0	6.0	0

**Table 4:** Choice of provider and days lost to productivity

Variables	N	Total days lost	Mean days lost	SD	Median	iqr (25th–75th percentile)
Chemical seller shop	165	644	3.9	4.1	3.0	0–7.0
CHPS	15	58.5	3.9	3.9	3.0	1.0–6.0
Private clinic	92	312.8	3.4	4.1	2.0	0–5.0
Drug peddler	41	168.1	4.1	4.3	3.0	0–7.0
Herbalist	7	48.3	6.9	6.4	3.0	2.0–14
Health centre	40	220	5.5	4.5	5.0	1.5–9
Hospital	108	529.2	4.9	4.3	4.0	0–7.0
Pharmacy	13	48.1	3.7	4.7	2.0	0–4.0
Total	481	2020.2	4.2	4.3	3.0	0–7.0

### Total cost of treating fever

Total direct cost was found to be GHS 4708.38 (US $3097.62) [with a mean cost per fever case of GHS 4.2 (US$ 2.76)]. Total indirect cost (productivity losses) was GHS 20,293.7 (US$ 13,351.12) [with a mean cost per fever case of GHS 18.0 (US$ 11.84)]. The overall total cost of treating fever for the entire study sample was GHS 25,002.04 (US$ 16,448.71) [with mean cost per fever case of GHS 22.2 (US$ 14.61)]. The highest cost was borne by the households who visited hospitals [total cost: GHS 7702.59 (US$ 5067.49); with mean cost per fever case of GHS 34.9 (US$ 22.96)]. Even though the total economic cost was lowest for those who visited herbalists [total cost: GHS 349 (US$ 229.61)] (Table 5).

**Table 5:** Direct, indirect and total economic costs of fever

Variables	Total cost GHS	Mean cost per patient GHS	SD GHS	Median GHS	iqr (25th–75th percentile) GHS
Direct cost					
All direct cost	4708.4	4.2	10.2	1.5	0.2–4.0
Medical costs	2110.4	1.9	7.9	0	0
Non-medical cost	1596.7	1.4	3.7	0	0–1.9
Direct cost of self-treatment	1001.3	0.9	2.4	0	0–1.0
Indirect costs					
All indirect cost	20,293.7	18.0	32.4	8.0	0–21.4
Indirect cost by occupation					
Farmers	6188.6	24.3	23.0	20.0	7.5–35.0
Formal sector employees	5,54.1	20.5	29.0	5.0	0–40.0
Traders	4187.4	46.5	70.9	20.5	0–60.0
Artisans	913.5	36.5	45.2	20.0	0–50.0
Unemployed	77	8.6	16.2	0	0–10.0
Under 18 years	8373.1	11.1	19.8	4.0	0–15.0
Total cost	25,002.0	22.2	35.7	11.0	2.0–28.0
Cost by health facility attended					
Chemical shop	6381.6	17.9	29.4	7.6	2.0–24.0
CHPS	635.1	14.1	15.4	9.6	5.0–15.0
Clinic	4608.1	22.8	41.8	8.0	0.7–30.0
Drug peddler	1147.9	12.8	22.3	4.0	1.0–15.0
Herbalist	349.0	34.9	46.9	12.5	5.0–67.0
Health centre	3456.4	24.0	31.7	15.0	4.3–30.0
Hospital	7702.6	30.1	43.0	18.9	5.7–37.4
Pharmacy	721.8	30.0	48.6	10.8	3.0–30.5

Exchange rate (as at 30th September 2011): $1 is equivalent to GHS 1.52 [27]

Distribution of costs according to wealth quintiles showed that the total cost as well as the mean cost per participant was lowest for those in the ‘highest’ quintile [total cost: GHS 3868.92; mean cost per fever case: GHS19.9 (CI 14.0–25.9)] and highest for those in the ‘middle’ quintiles [total cost: GHS 6686.28; mean cost per fever case: GHS 24.1]. Those in the highest quintile spent a lower proportion of their income on cost of fever care compared to those in the lowest quintile (Table 6).

**Table 6:** Economic burden of fever cost on household’s income

Wealth quintile groupings	Households annual monthly income (GHS)	Mean economic cost of fever (GHS)	Share of cost on income (%)
Highest	3868.9	19.9	13.8
Fourth	4680.0	21.0	19.1
Middle	6686.3	24.1	36.2
Second	4296.8	20.4	31.9
Lowest	5469.5	24.4	54.5

Exchange rate (as at 30th September 2011): GH$1 is equivalent to GHS 1.52 [27]

## Discussion

The overall mean cost of fever was GHS 22.2 ($14.6). This is high given that the study area is predominantly rural with relatively poor inhabitants. The results also showed that the cost was higher for those in the lower wealth quintile quintiles compared to those in the higher wealth quintiles. This trend can have a number of effects on the poor. For example, it can prevent the poor from seeking care when they have a fever episode. This is especially true if they are not members of the NHIS and will have to pay out of their pockets for health care. High cost combined with out-of-pocket payments can lead to catastrophic payments by the poor which can further cause the poor to be poorer and also push those above the poverty line into impoverishment [28–30].

The study findings further showed that the cost was highest for those who attended hospitals [GHS 30.1]. Direct medical cost was identified to form the highest component of the direct costs incurred by households and this was influenced mostly by the cost of hospitalization. This was probably because patients attended the hospitals when their fever was severe and required more days for treatment and extensive treatment and services including hospitalization in most cases. The average number of days lost due to fever by patients (4.2 days) and days absent from school (2.4 days) were comparable to findings from other settings. For instance, in related studies in Ghana and Ethiopia [10, 12, 31], the number of days lost due to malaria by patients and the lost school days ranged between three to nine days [6, 10, 11]. The number of days lost, to some extent influenced the productivity losses for the respondents. Productivity losses are worrying due to the fact that most inhabitants in the study area are farmers and peak malaria season coincides with the rainy seasons. Days lost to fever affect household’s productivity and this in effect affects household’s income and also the national economy.

Unlike other studies where households mostly borrowed money or sold their assets to cover the cost of health care [32, 33], the majority of respondents in this study paid for care of febrile illness using their health insurance or cash. Respondents who paid out-of-pocket were mostly those in the lowest quintile. Out-of-pocket payment as a health financing mechanism is known to be regressive [34] rather than progressive: as poorer households bear a higher burden of the cost compared to the richer households. The economic burden of the cost of fever presented in this study was higher on those in the lower wealth quintiles compared to those in the higher wealth quintiles as they used a greater share of their monthly mean income to cover the cost of treating their fever.

Household members sought care from different sources when they had a fever and majority of the respondents sought care from the formal health care facilities (private clinics, hospitals, health centres, CHPs compounds) [6, 35]. The result suggests that treatment-seeking behaviour has changed compared to the past decade when households resorted to home treatments [6, 35]. This positive trend of care-seeking behaviour can help minimize self-medication and misguided traditional treatment and their associated complications and costs. The encouraging trend of seeking care from formal health care facilities could be because majority of health facilities are health insurance service providers while community based licensed chemical sellers are not. It is, therefore, anticipated that an increase in the National Health Insurance Scheme (NHIS) coverage in the area could positively influence households to seek care in formal health care facilities. The positive impact of NHIS on formal health care attendance is corroborated in other studies [36–38].

## Limitations

Despite these findings, there are some limitations to this study. Firstly, the study used self-reported fever to indicate malaria. Although not all fevers are malaria, in Ghana fever is mostly associated with malaria. Secondly, the costs presented in this study may not be transferable to other contexts where health-seeking behaviour, insurance coverage, and occupation differ from those in rural Ghana. Finally, there was recall bias as participants were asked to recall over a period of 2 weeks. There is possibility of either underestimation or overestimation as they reported expenditure verbally without producing receipts.

## Conclusion

The total cost of fever episode of GHS 22.2 (US$14.61) is quite high considering the poverty level in the middle belt of Ghana especially the study setting were majority of the households live below the poverty level. The burden of fever falls disproportionately on poor households as close to five working days are lost due to fever which may further push them into poverty. The NHIS has positively influenced health-seeking behaviours and reduced the financial burden of seeking care to those that are insured.
